# (1,2,3,4-Tetra­hydro­isoquinoline-2-carbo­dithio­ato-κ^2^ *S*,*S*′)(thio­cyanato-κ*N*)(tri­phenyl­phosphane)nickel(II)

**DOI:** 10.1107/S1600536811050550

**Published:** 2011-11-30

**Authors:** P. Valarmathi, S. Thirumaran, S. Selvanayagam

**Affiliations:** a Department of Chemistry, Annamalai University, Annamalainagar 608 002, India; b Department of Physics, Kalasalingam University, Krishnankoil 626 126, India

## Abstract

The Ni^II^ atom in the mononuclear title compound, [Ni(C_10_H_10_NS_2_)(NCS)(C_18_H_15_P)], exists within a S_2_PN donor set that defines a distorted square-planar geometry. A significant asymmetry in the Ni—S bond lengths support the less effective *trans* effect of SCN^−^ over PPh_3_.

## Related literature

For general background to dithio­carbamates and their bio­logical activity, see: Gunay *et al.* (1999 ▶); Hogarth (2005 ▶); Ozkirimli *et al.* (2005 ▶). Nickel complexes of phosphine ligands have been studied for their anti­cancer activity, see: Jarret *et al.* (1993 ▶). Nickel(II) dithio­carbamates can react with Lewis bases such as phosphines as well as hard bases such as nitro­genous ligands, see: Srinivasan *et al.* (2009 ▶); Travnicek *et al.* (2008 ▶). For the preparation of the title compound, see: Valarmathi *et al.* (2011 ▶).
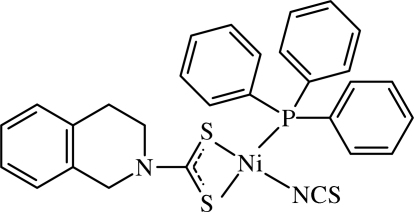


## Experimental

### 

#### Crystal data

[Ni(C_10_H_10_NS_2_)(NCS)(C_18_H_15_P)]
*M* *_r_* = 587.37
Monoclinic, 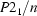

*a* = 13.7981 (4) Å
*b* = 13.1429 (4) Å
*c* = 14.9447 (4) Å
β = 91.693 (2)°
*V* = 2708.99 (13) Å^3^
*Z* = 4
Mo *K*α radiation
μ = 1.03 mm^−1^
*T* = 292 K
0.25 × 0.20 × 0.15 mm

#### Data collection

Bruker Kappa APEXII CCD diffractometer
Absorption correction: multi-scan (*SADABS*; Bruker 1999 ▶) *T*_min_ = 0.783, *T*_max_ = 0.861
33862 measured reflections
7287 independent reflections
5132 reflections with *I* > 2σ(*I*)
*R*_int_ = 0.035

#### Refinement

*R*[*F* ^2^ > 2σ(*F* ^2^)] = 0.042
*wR*(*F* ^2^) = 0.123
*S* = 1.03
7287 reflections
325 parameters
H-atom parameters constrained
Δρ_max_ = 0.55 e Å^−3^
Δρ_min_ = −0.30 e Å^−3^

### 

Data collection: *APEX2* (Bruker, 2004 ▶); cell refinement: *SAINT* (Bruker, 2004 ▶); data reduction: *SAINT* and *XPREP* (Bruker, 2004 ▶); program(s) used to solve structure: *SIR92* (Altomare *et al.*, 1993 ▶); program(s) used to refine structure: *SHELXL97* (Sheldrick, 2008 ▶); molecular graphics: *ORTEP-3* (Farrugia, 1997 ▶) and *PLATON* (Spek, 2009 ▶); software used to prepare material for publication: *SHELXL97* and *PLATON*.

## Supplementary Material

Crystal structure: contains datablock(s) I, global. DOI: 10.1107/S1600536811050550/bt5724sup1.cif

Structure factors: contains datablock(s) I. DOI: 10.1107/S1600536811050550/bt5724Isup2.hkl

Additional supplementary materials:  crystallographic information; 3D view; checkCIF report

## Figures and Tables

**Table 1:** Selected bond lengths (Å)

N1—Ni1	1.867 (2)
P1—Ni1	2.1874 (6)
S1—Ni1	2.218 (1)
S2—Ni1	2.162 (1)

**Fig. 1.**
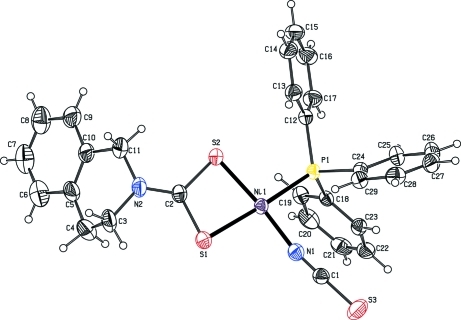
The molecular structure of the title compound, showing the atom-numbering scheme. Displacement ellipsoids are drawn at the 30% probability level

**Fig. 2.**
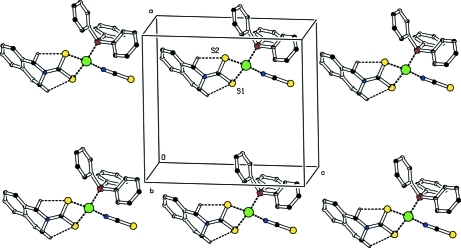
Molecular packing of the title compound, viewed along the b axis; H-bonds are shown as dashed lines. For the sake of clarity, H atoms, not involved in hydrogen bonds, have been omitted

## supplementary crystallographic information

### Comment

Dithiocarbamates are versatile ligands which have been shown to bind to all transition elements supporting a wide range of oxidation state (Hogarth, 2005). They have been shown to posses a broad spectrum of biological activities such as fungicidal (Ozkirimli *et al.*, 2005) and bactericidal (Gunay *et al.*, 1999). Nickel complexes of phosphine ligands have been studied for their anticancer activity (Jarret *et al.*, 1993). Nickel(II) dithiocarbamates are borderline acceptors and they can react with Lewis bases such as phosphines as well as hard bases such as nitrogenous ligands (Srinivasan *et al.*, 2009; Travnicek *et al.*, 2008). In view of these importance we have undertaken the crystal structure determination of the title compound, and the results are presented here.

The X-ray study confirmed the molecular structure and atomic connectivity for (I), as illustrated in Fig. 1.

The structure consists of distorted square planar metal coordination with NiS_2_PN chromophore.  Deviation of the plane from a perfect square is caused by the small bite angle subtended by the sulfur atoms of the chelating dithiocarbamate at the nickel atom. The Ni—S bond distances [2.218 (1) and 2.162 (1) Å, respectively] are significantly different, due to the different trans influences exerted by phosphine and NCS^-^.  PPh_3_ being a good π-acceptor has a greater trans influence and hence the Ni—S bond trans to P is longer than the one trans to NCS anion.

The shortening of Ni—P distance is due the strong back bonding in nickel atom. The C—P—C angles deviate appreciably from the normal tetrahedral angle due to the crowding of the phenyl rings.  The short Ni—N distance, 1.867 (2) Å, shows the effective bonding between the nickel atom and NCS^-^. The Ni—N—C angle 170.9 (2)° indicates deviation from the linearity and is due to steric compulsions of the bulky PPh_3_ group.

The C—S bond lengths are 1.707 (3) and 1.717 (3) Å which are shorter than the typical single bond value of 1.81 Å and longer than C=S distance of 1.69 Å, indicating partial double bond character.  The short thioureide C—N distance, 1.315 (3) Å indicates that the π-electron density is delocalised over the S_2_CN moiety and that this bond has partial double bond character.

In addition to the van der Waals interactions, the molecular structure is influenced only by intramolecular C—H···S hydrogen bonds involving sulphur atoms S1 and S2. (Fig. 2 and Table 1).

### Experimental

Compound (I) was prepared according to the literature procedure (Valarmathi *et al.*, 2011). Single crystals of (I) were obtained by slow evaporation of dichloromethane and ethanol (2:1) solution of (I) at room temperature.

### Refinement

H atoms were placed in idealized positions and allowed to ride on their parent atoms, with C—H distances of 0.93-0.97 Å, and Uiso(H) = 1.2U_eq_(C) for H atoms.

### Crystal data

**Table:**

[Ni(C_10_H_10_NS_2_)(NCS)(C_18_H_15_P)]	*F*(000) = 1216
*M**_r_* = 587.37	*D*_x_ = 1.440 Mg m^−^^3^
Monoclinic, *P*2_1_/*n*	Mo *K*α radiation, λ = 0.71073 Å
Hall symbol: -P 2yn	Cell parameters from 8723 reflections
*a* = 13.7981 (4) Å	θ = 2.5–26.3°
*b* = 13.1429 (4) Å	µ = 1.03 mm^−^^1^
*c* = 14.9447 (4) Å	*T* = 292 K
β = 91.693 (2)°	Block, pale-yellow
*V* = 2708.99 (13) Å^3^	0.25 × 0.20 × 0.15 mm
*Z* = 4	

### Data collection

**Table:**

Bruker KAPPA APEXII CCD diffractometer	7287 independent reflections
Radiation source: fine-focus sealed tube	5132 reflections with *I* > 2σ(*I*)
graphite	*R*_int_ = 0.035
ω and φ scan	θ_max_ = 29.2°, θ_min_ = 2.0°
Absorption correction: multi-scan (*SADABS*; Bruker 1999)	*h* = −18→17
*T*_min_ = 0.783, *T*_max_ = 0.861	*k* = −11→17
33862 measured reflections	*l* = −20→20

### Refinement

**Table:**

Refinement on *F*^2^	Primary atom site location: structure-invariant direct methods
Least-squares matrix: full	Secondary atom site location: difference Fourier map
*R*[*F*^2^ > 2σ(*F*^2^)] = 0.042	Hydrogen site location: inferred from neighbouring sites
*wR*(*F*^2^) = 0.123	H-atom parameters constrained
*S* = 1.03	*w* = 1/[σ^2^(*F*_o_^2^) + (0.0574*P*)^2^ + 1.2033*P*] where *P* = (*F*_o_^2^ + 2*F*_c_^2^)/3
7287 reflections	(Δ/σ)_max_ = 0.001
325 parameters	Δρ_max_ = 0.55 e Å^−^^3^
0 restraints	Δρ_min_ = −0.30 e Å^−^^3^

### Special details

**Table:**

Geometry. All esds (except the esd in the dihedral angle between two l.s. planes) are estimated using the full covariance matrix. The cell esds are taken into account individually in the estimation of esds in distances, angles and torsion angles; correlations between esds in cell parameters are only used when they are defined by crystal symmetry. An approximate (isotropic) treatment of cell esds is used for estimating esds involving l.s. planes.
Refinement. Refinement of F^2^ against ALL reflections. The weighted R-factor wR and goodness of fit S are based on F^2^, conventional R-factors R are based on F, with F set to zero for negative F^2^. The threshold expression of F^2^ > 2sigma(F^2^) is used only for calculating R-factors(gt) etc. and is not relevant to the choice of reflections for refinement. R-factors based on F^2^ are statistically about twice as large as those based on F, and R- factors based on ALL data will be even larger.

### Fractional atomic coordinates and isotropic or equivalent isotropic displacement parameters (Å^2^)

**Table:**

	*x*	*y*	*z*	*U*_iso_*/*U*_eq_	
C1	0.67037 (18)	0.52602 (19)	0.77990 (18)	0.0474 (6)	
C2	0.70333 (19)	0.61484 (19)	0.43746 (18)	0.0486 (6)	
C3	0.5892 (3)	0.5795 (3)	0.3133 (2)	0.0739 (9)	
H3A	0.5591	0.6295	0.2737	0.089*	
H3B	0.5448	0.5654	0.3609	0.089*	
C4	0.6072 (3)	0.4837 (3)	0.2622 (3)	0.0850 (11)	
H4A	0.6227	0.4289	0.3036	0.102*	
H4B	0.5490	0.4650	0.2280	0.102*	
C5	0.6874 (2)	0.4983 (3)	0.2015 (2)	0.0694 (9)	
C6	0.6980 (4)	0.4273 (3)	0.1283 (3)	0.0919 (12)	
H6	0.6541	0.3740	0.1207	0.110*	
C7	0.7711 (4)	0.4377 (4)	0.0712 (3)	0.0969 (13)	
H7	0.7773	0.3920	0.0242	0.116*	
C8	0.8378 (3)	0.5175 (4)	0.0825 (3)	0.0950 (13)	
H8	0.8875	0.5249	0.0424	0.114*	
C9	0.8305 (3)	0.5845 (3)	0.1519 (2)	0.0777 (10)	
H9	0.8755	0.6365	0.1602	0.093*	
C10	0.7549 (2)	0.5731 (2)	0.20951 (19)	0.0592 (7)	
C11	0.7468 (3)	0.6536 (3)	0.2850 (2)	0.0742 (9)	
H11A	0.8102	0.6644	0.3131	0.089*	
H11B	0.7250	0.7176	0.2593	0.089*	
C12	1.00159 (18)	0.65116 (18)	0.63240 (16)	0.0424 (5)	
C13	0.9915 (2)	0.7503 (2)	0.60153 (18)	0.0518 (6)	
H13	0.9327	0.7838	0.6071	0.062*	
C14	1.0679 (2)	0.7995 (2)	0.5627 (2)	0.0631 (8)	
H14	1.0608	0.8663	0.5430	0.076*	
C15	1.1541 (2)	0.7499 (3)	0.5532 (2)	0.0698 (9)	
H15	1.2054	0.7827	0.5263	0.084*	
C16	1.1653 (2)	0.6520 (3)	0.5833 (2)	0.0707 (9)	
H16	1.2240	0.6185	0.5765	0.085*	
C17	1.0890 (2)	0.6025 (2)	0.6240 (2)	0.0580 (7)	
H17	1.0972	0.5366	0.6455	0.070*	
C18	0.92192 (16)	0.46836 (17)	0.71702 (16)	0.0401 (5)	
C19	0.9523 (2)	0.3997 (2)	0.65279 (19)	0.0526 (6)	
H19	0.9652	0.4223	0.5954	0.063*	
C20	0.9633 (2)	0.2978 (2)	0.6743 (2)	0.0663 (8)	
H20	0.9850	0.2525	0.6315	0.080*	
C21	0.9426 (2)	0.2629 (2)	0.7583 (2)	0.0632 (8)	
H21	0.9508	0.1945	0.7725	0.076*	
C22	0.9100 (2)	0.3294 (2)	0.8207 (2)	0.0587 (7)	
H22	0.8946	0.3056	0.8771	0.070*	
C23	0.89970 (19)	0.4318 (2)	0.80110 (18)	0.0492 (6)	
H23	0.8778	0.4762	0.8445	0.059*	
C24	0.89695 (17)	0.67053 (17)	0.79026 (15)	0.0404 (5)	
C25	0.9742 (2)	0.6615 (2)	0.85052 (18)	0.0534 (6)	
H25	1.0243	0.6165	0.8390	0.064*	
C26	0.9775 (2)	0.7188 (2)	0.9275 (2)	0.0671 (8)	
H26	1.0290	0.7114	0.9685	0.080*	
C27	0.9048 (3)	0.7866 (2)	0.9437 (2)	0.0658 (8)	
H27	0.9073	0.8254	0.9958	0.079*	
C28	0.8290 (2)	0.7977 (2)	0.8845 (2)	0.0619 (7)	
H28	0.7803	0.8445	0.8958	0.074*	
C29	0.82414 (19)	0.7394 (2)	0.80687 (18)	0.0503 (6)	
H29	0.7721	0.7468	0.7664	0.060*	
N1	0.69817 (16)	0.54668 (18)	0.71207 (15)	0.0518 (5)	
N2	0.67965 (18)	0.6210 (2)	0.35176 (15)	0.0612 (6)	
P1	0.89629 (4)	0.59881 (4)	0.68659 (4)	0.03763 (14)	
S1	0.63111 (5)	0.56870 (6)	0.51886 (5)	0.05756 (19)	
S2	0.81349 (5)	0.64882 (6)	0.48486 (4)	0.05379 (18)	
S3	0.63080 (8)	0.50024 (8)	0.87781 (6)	0.0838 (3)	
Ni1	0.75893 (2)	0.59313 (2)	0.60984 (2)	0.04025 (10)	

### Atomic displacement parameters (Å^2^)

**Table:**

	*U*^11^	*U*^22^	*U*^33^	*U*^12^	*U*^13^	*U*^23^
C1	0.0428 (14)	0.0467 (13)	0.0524 (16)	−0.0003 (10)	−0.0034 (11)	0.0053 (11)
C2	0.0488 (15)	0.0510 (14)	0.0455 (14)	0.0054 (11)	−0.0073 (11)	0.0023 (11)
C3	0.068 (2)	0.098 (3)	0.0535 (18)	0.0035 (17)	−0.0239 (16)	−0.0040 (16)
C4	0.069 (2)	0.098 (3)	0.086 (3)	−0.0164 (19)	−0.0205 (19)	−0.003 (2)
C5	0.067 (2)	0.078 (2)	0.0623 (19)	−0.0082 (16)	−0.0242 (16)	0.0088 (16)
C6	0.109 (3)	0.081 (2)	0.084 (3)	0.013 (2)	−0.023 (2)	−0.011 (2)
C7	0.106 (3)	0.111 (3)	0.072 (3)	0.034 (3)	−0.020 (2)	−0.023 (2)
C8	0.091 (3)	0.121 (3)	0.073 (2)	0.033 (3)	−0.014 (2)	−0.006 (2)
C9	0.070 (2)	0.100 (3)	0.063 (2)	0.0029 (18)	−0.0101 (17)	0.0145 (18)
C10	0.0552 (17)	0.0711 (18)	0.0505 (16)	0.0061 (13)	−0.0122 (13)	0.0131 (13)
C11	0.087 (2)	0.088 (2)	0.0470 (17)	−0.0120 (18)	−0.0133 (16)	0.0092 (15)
C12	0.0427 (13)	0.0472 (13)	0.0371 (12)	−0.0033 (10)	−0.0012 (10)	−0.0015 (10)
C13	0.0558 (16)	0.0490 (14)	0.0506 (15)	−0.0033 (12)	0.0039 (12)	0.0004 (11)
C14	0.083 (2)	0.0525 (15)	0.0543 (17)	−0.0181 (15)	0.0068 (15)	−0.0016 (13)
C15	0.067 (2)	0.082 (2)	0.0611 (19)	−0.0297 (17)	0.0171 (15)	−0.0127 (16)
C16	0.0481 (17)	0.083 (2)	0.081 (2)	−0.0028 (15)	0.0127 (15)	−0.0028 (18)
C17	0.0484 (16)	0.0613 (16)	0.0645 (18)	0.0008 (12)	0.0058 (13)	0.0031 (13)
C18	0.0359 (12)	0.0424 (12)	0.0416 (12)	0.0001 (9)	−0.0062 (9)	−0.0004 (10)
C19	0.0547 (16)	0.0523 (15)	0.0507 (15)	0.0004 (12)	−0.0032 (12)	−0.0057 (12)
C20	0.0629 (19)	0.0477 (15)	0.088 (2)	0.0046 (13)	−0.0062 (16)	−0.0211 (15)
C21	0.0542 (17)	0.0381 (13)	0.096 (2)	−0.0036 (11)	−0.0123 (16)	0.0037 (15)
C22	0.0560 (17)	0.0531 (15)	0.0664 (18)	−0.0091 (12)	−0.0106 (14)	0.0164 (14)
C23	0.0524 (15)	0.0490 (13)	0.0458 (14)	−0.0008 (11)	−0.0034 (11)	0.0031 (11)
C24	0.0448 (13)	0.0396 (11)	0.0369 (12)	0.0000 (9)	0.0003 (10)	0.0002 (9)
C25	0.0569 (16)	0.0524 (14)	0.0499 (15)	0.0060 (12)	−0.0120 (12)	−0.0062 (12)
C26	0.079 (2)	0.0700 (19)	0.0515 (17)	−0.0049 (16)	−0.0184 (15)	−0.0068 (14)
C27	0.089 (2)	0.0596 (17)	0.0493 (16)	−0.0127 (16)	0.0071 (16)	−0.0168 (14)
C28	0.0659 (19)	0.0561 (16)	0.0646 (18)	0.0009 (13)	0.0178 (15)	−0.0133 (14)
C29	0.0476 (15)	0.0514 (14)	0.0519 (15)	0.0038 (11)	0.0013 (12)	−0.0030 (11)
N1	0.0451 (12)	0.0603 (13)	0.0498 (13)	0.0018 (10)	−0.0036 (10)	0.0028 (11)
N2	0.0602 (15)	0.0798 (16)	0.0428 (13)	0.0006 (12)	−0.0109 (11)	0.0018 (11)
P1	0.0381 (3)	0.0410 (3)	0.0336 (3)	0.0027 (2)	−0.0021 (2)	0.0014 (2)
S1	0.0445 (4)	0.0752 (5)	0.0522 (4)	−0.0037 (3)	−0.0108 (3)	0.0107 (3)
S2	0.0529 (4)	0.0690 (4)	0.0390 (3)	−0.0097 (3)	−0.0059 (3)	0.0059 (3)
S3	0.1015 (7)	0.0936 (7)	0.0572 (5)	−0.0079 (5)	0.0185 (5)	0.0166 (4)
Ni1	0.03948 (18)	0.04412 (17)	0.03685 (17)	0.00331 (12)	−0.00393 (12)	0.00315 (12)

### Geometric parameters (Å, °)

**Table:**

C1—N1	1.128 (3)	C15—C16	1.370 (5)
C1—S3	1.613 (3)	C15—H15	0.9300
C2—N2	1.315 (3)	C16—C17	1.392 (4)
C2—S1	1.707 (3)	C16—H16	0.9300
C2—S2	1.717 (3)	C17—H17	0.9300
C3—N2	1.464 (4)	C18—C23	1.388 (3)
C3—C4	1.497 (5)	C18—C19	1.391 (3)
C3—H3A	0.9700	C18—P1	1.806 (2)
C3—H3B	0.9700	C19—C20	1.385 (4)
C4—C5	1.464 (5)	C19—H19	0.9300
C4—H4A	0.9700	C20—C21	1.374 (5)
C4—H4B	0.9700	C20—H20	0.9300
C5—C10	1.357 (4)	C21—C22	1.364 (4)
C5—C6	1.449 (5)	C21—H21	0.9300
C6—C7	1.347 (6)	C22—C23	1.383 (4)
C6—H6	0.9300	C22—H22	0.9300
C7—C8	1.402 (6)	C23—H23	0.9300
C7—H7	0.9300	C24—C25	1.380 (3)
C8—C9	1.366 (5)	C24—C29	1.381 (3)
C8—H8	0.9300	C24—P1	1.813 (2)
C9—C10	1.380 (5)	C25—C26	1.375 (4)
C9—H9	0.9300	C25—H25	0.9300
C10—C11	1.553 (5)	C26—C27	1.368 (5)
C11—N2	1.447 (4)	C26—H26	0.9300
C11—H11A	0.9700	C27—C28	1.358 (4)
C11—H11B	0.9700	C27—H27	0.9300
C12—C17	1.374 (4)	C28—C29	1.390 (4)
C12—C13	1.387 (4)	C28—H28	0.9300
C12—P1	1.819 (2)	C29—H29	0.9300
C13—C14	1.380 (4)	N1—Ni1	1.867 (2)
C13—H13	0.9300	P1—Ni1	2.1874 (6)
C14—C15	1.367 (5)	S1—Ni1	2.218 (1)
C14—H14	0.9300	S2—Ni1	2.162 (1)
			
N1—C1—S3	178.2 (3)	C12—C17—H17	120.0
N2—C2—S1	125.6 (2)	C16—C17—H17	120.0
N2—C2—S2	125.3 (2)	C23—C18—C19	118.6 (2)
S1—C2—S2	109.07 (14)	C23—C18—P1	120.66 (19)
N2—C3—C4	111.2 (3)	C19—C18—P1	120.16 (19)
N2—C3—H3A	109.4	C20—C19—C18	120.0 (3)
C4—C3—H3A	109.4	C20—C19—H19	120.0
N2—C3—H3B	109.4	C18—C19—H19	120.0
C4—C3—H3B	109.4	C21—C20—C19	120.7 (3)
H3A—C3—H3B	108.0	C21—C20—H20	119.6
C5—C4—C3	110.2 (3)	C19—C20—H20	119.6
C5—C4—H4A	109.6	C22—C21—C20	119.5 (3)
C3—C4—H4A	109.6	C22—C21—H21	120.3
C5—C4—H4B	109.6	C20—C21—H21	120.3
C3—C4—H4B	109.6	C21—C22—C23	120.9 (3)
H4A—C4—H4B	108.1	C21—C22—H22	119.6
C10—C5—C6	116.6 (4)	C23—C22—H22	119.6
C10—C5—C4	124.8 (3)	C22—C23—C18	120.3 (3)
C6—C5—C4	118.6 (3)	C22—C23—H23	119.9
C7—C6—C5	120.5 (4)	C18—C23—H23	119.9
C7—C6—H6	119.8	C25—C24—C29	119.4 (2)
C5—C6—H6	119.8	C25—C24—P1	119.81 (19)
C6—C7—C8	120.0 (4)	C29—C24—P1	120.62 (19)
C6—C7—H7	120.0	C26—C25—C24	120.3 (3)
C8—C7—H7	120.0	C26—C25—H25	119.8
C9—C8—C7	120.7 (4)	C24—C25—H25	119.8
C9—C8—H8	119.6	C27—C26—C25	119.9 (3)
C7—C8—H8	119.6	C27—C26—H26	120.0
C8—C9—C10	118.5 (4)	C25—C26—H26	120.0
C8—C9—H9	120.8	C28—C27—C26	120.6 (3)
C10—C9—H9	120.8	C28—C27—H27	119.7
C5—C10—C9	123.6 (3)	C26—C27—H27	119.7
C5—C10—C11	119.6 (3)	C27—C28—C29	120.1 (3)
C9—C10—C11	116.8 (3)	C27—C28—H28	119.9
N2—C11—C10	111.1 (3)	C29—C28—H28	119.9
N2—C11—H11A	109.4	C24—C29—C28	119.6 (3)
C10—C11—H11A	109.4	C24—C29—H29	120.2
N2—C11—H11B	109.4	C28—C29—H29	120.2
C10—C11—H11B	109.4	C1—N1—Ni1	170.9 (2)
H11A—C11—H11B	108.0	C2—N2—C11	123.0 (3)
C17—C12—C13	119.1 (2)	C2—N2—C3	122.9 (3)
C17—C12—P1	125.4 (2)	C11—N2—C3	113.3 (2)
C13—C12—P1	115.43 (19)	C18—P1—C24	106.40 (11)
C14—C13—C12	120.6 (3)	C18—P1—C12	108.54 (11)
C14—C13—H13	119.7	C24—P1—C12	101.53 (11)
C12—C13—H13	119.7	C18—P1—Ni1	105.06 (8)
C15—C14—C13	119.9 (3)	C24—P1—Ni1	116.63 (8)
C15—C14—H14	120.1	C12—P1—Ni1	118.07 (8)
C13—C14—H14	120.1	C2—S1—Ni1	85.13 (9)
C14—C15—C16	120.2 (3)	C2—S2—Ni1	86.67 (9)
C14—C15—H15	119.9	N1—Ni1—S2	173.69 (7)
C16—C15—H15	119.9	N1—Ni1—P1	89.15 (7)
C15—C16—C17	120.2 (3)	S2—Ni1—P1	97.07 (3)
C15—C16—H16	119.9	N1—Ni1—S1	94.94 (7)
C17—C16—H16	119.9	S2—Ni1—S1	79.06 (3)
C12—C17—C16	120.0 (3)	P1—Ni1—S1	170.83 (3)
			
N2—C3—C4—C5	−48.3 (4)	C10—C11—N2—C2	125.0 (3)
C3—C4—C5—C10	19.4 (5)	C10—C11—N2—C3	−45.3 (4)
C3—C4—C5—C6	−162.1 (3)	C4—C3—N2—C2	−105.5 (4)
C10—C5—C6—C7	−1.1 (5)	C4—C3—N2—C11	64.7 (4)
C4—C5—C6—C7	−179.7 (4)	C23—C18—P1—C24	26.0 (2)
C5—C6—C7—C8	0.3 (6)	C19—C18—P1—C24	−162.5 (2)
C6—C7—C8—C9	0.9 (6)	C23—C18—P1—C12	134.6 (2)
C7—C8—C9—C10	−1.3 (5)	C19—C18—P1—C12	−53.9 (2)
C6—C5—C10—C9	0.7 (5)	C23—C18—P1—Ni1	−98.25 (19)
C4—C5—C10—C9	179.2 (3)	C19—C18—P1—Ni1	73.2 (2)
C6—C5—C10—C11	178.6 (3)	C25—C24—P1—C18	50.6 (2)
C4—C5—C10—C11	−2.9 (5)	C29—C24—P1—C18	−133.8 (2)
C8—C9—C10—C5	0.5 (5)	C25—C24—P1—C12	−62.8 (2)
C8—C9—C10—C11	−177.4 (3)	C29—C24—P1—C12	112.7 (2)
C5—C10—C11—N2	15.0 (4)	C25—C24—P1—Ni1	167.41 (18)
C9—C10—C11—N2	−167.0 (3)	C29—C24—P1—Ni1	−17.1 (2)
C17—C12—C13—C14	0.1 (4)	C17—C12—P1—C18	−5.7 (3)
P1—C12—C13—C14	177.2 (2)	C13—C12—P1—C18	177.39 (19)
C12—C13—C14—C15	1.0 (4)	C17—C12—P1—C24	106.1 (2)
C13—C14—C15—C16	−0.9 (5)	C13—C12—P1—C24	−70.8 (2)
C14—C15—C16—C17	−0.2 (5)	C17—C12—P1—Ni1	−125.0 (2)
C13—C12—C17—C16	−1.3 (4)	C13—C12—P1—Ni1	58.1 (2)
P1—C12—C17—C16	−178.1 (2)	N2—C2—S1—Ni1	176.0 (3)
C15—C16—C17—C12	1.4 (5)	S2—C2—S1—Ni1	−2.32 (12)
C23—C18—C19—C20	−2.4 (4)	N2—C2—S2—Ni1	−176.0 (2)
P1—C18—C19—C20	−174.1 (2)	S1—C2—S2—Ni1	2.37 (12)
C18—C19—C20—C21	1.4 (4)	C1—N1—Ni1—S2	−139.3 (12)
C19—C20—C21—C22	0.6 (4)	C1—N1—Ni1—P1	30.9 (14)
C20—C21—C22—C23	−1.5 (4)	C1—N1—Ni1—S1	−157.3 (14)
C21—C22—C23—C18	0.4 (4)	C2—S2—Ni1—N1	−20.1 (7)
C19—C18—C23—C22	1.6 (4)	C2—S2—Ni1—P1	169.83 (9)
P1—C18—C23—C22	173.2 (2)	C2—S2—Ni1—S1	−1.76 (9)
C29—C24—C25—C26	1.6 (4)	C18—P1—Ni1—N1	64.00 (11)
P1—C24—C25—C26	177.2 (2)	C24—P1—Ni1—N1	−53.51 (11)
C24—C25—C26—C27	−1.3 (5)	C12—P1—Ni1—N1	−174.89 (12)
C25—C26—C27—C28	0.2 (5)	C18—P1—Ni1—S2	−117.09 (9)
C26—C27—C28—C29	0.6 (5)	C24—P1—Ni1—S2	125.41 (9)
C25—C24—C29—C28	−0.8 (4)	C12—P1—Ni1—S2	4.03 (9)
P1—C24—C29—C28	−176.4 (2)	C18—P1—Ni1—S1	−52.7 (2)
C27—C28—C29—C24	−0.3 (4)	C24—P1—Ni1—S1	−170.20 (18)
S3—C1—N1—Ni1	46 (9)	C12—P1—Ni1—S1	68.4 (2)
S1—C2—N2—C11	−175.1 (2)	C2—S1—Ni1—N1	179.78 (11)
S2—C2—N2—C11	3.0 (4)	C2—S1—Ni1—S2	1.77 (9)
S1—C2—N2—C3	−5.8 (4)	C2—S1—Ni1—P1	−63.9 (2)
S2—C2—N2—C3	172.3 (2)		
